# A New Species of *Pachytriton* (Amphibia: Caudata: Salamandridae) from Anhui, China

**DOI:** 10.3390/ani15203018

**Published:** 2025-10-17

**Authors:** Zhirong He, Siyu Wu, Shanqing Wang, Li Ma, Na Zhao, Xiaobing Wu, Supen Wang

**Affiliations:** 1 The Anhui Provincial Key Laboratory of Biodiversity Conservation and Ecological Security in the Yangtze River Basin, College of Life Sciences, Anhui Normal University, Wuhu 241000, China; zhirong@ahnu.edu.cn (Z.H.); 19855304469@163.com (S.W.);; 2 Shexian Management Station of Anhui Qingliangfeng National Nature Reserve, Huangshan 245200, China; 13399591156@163.com; 3 College of Ecology, Lishui University, Lishui 323000, China; lma2023@126.com

**Keywords:** *Pachytriton cheni* sp. nov., southeast Chinese hilly area, morphology, molecular phylogeny, multispecies coalescent model, species delimitation, taxonomy, biodiversity

## Abstract

China is a global hotspot for amphibian biodiversity, yet under-explored montane regions harbor undiscovered cryptic species. Using integrative taxonomy, we describe a new salamandrid species, *Pachytriton cheni* sp. nov., from Qingliangfeng Nature Reserve, Anhui. Phylogenetic analyses of mitochondrial (*ND2*, *cytb*) and nuclear (*RAG1*, *POMC*) genes strongly support it as a monophyletic sister lineage to *P. granulosus*, with significant mitochondrial *p*-distances (4.39–10.22%) and unique nuclear haplotypes. Bayes factor species delimitation under the multispecies coalescent model (MSC) decisively rejects conspecificity with *P. granulosus* (2lnBF = 24.52). Morphologically, it is diagnosed by its small size; oval, narrow head (length > width); nearly black dorsum lacking bright orange spots; smooth skin; occipital V-shaped ridge; orange-red abdomen with brown markings; prominent neck folds; and minimal digit gap when limbs are adpressed. This discovery increases *Pachytriton* species to ten, highlights high-elevation montane ecosystems as key biodiversity refuges in East China, and underscores the need for further surveys to clarify the genus’s phylogeny.

## Simple Summary

China is home to many unique amphibians, but some species remain undiscovered in remote mountain areas. In this study, scientists identified a new species of salamander from Qingliangfeng Nature Reserve in Anhui Province. The researchers used molecular and morphological analysis to confirm that this salamander is different from any known species. The new salamander is small, with a body length of 57.1–69.1 mm for males and 63.9–68.7 mm for females. It has a narrow head, smooth black skin on its back, and an orange-red belly with brown markings. A distinctive V-shaped ridge on the back of its head and prominent neck folds further distinguish it from similar species. DNA tests showed clear genetic differences from related salamanders, confirming it as a separate species. This discovery increases the number of known salamander species in this group to eleven, highlighting the rich biodiversity of China’s mountain ecosystems. As a protected species living in a specific high-elevation habitat, this new salamander needs special conservation attention. The findings contribute to our understanding of biodiversity and provide valuable information for developing effective conservation strategies to protect this unique species and its fragile mountain stream habitat.

## 1. Introduction

China has prioritized biodiversity conservation as a key national strategy, aligning with the global vision of a shared future for all life. Amphibians, as the most threatened group of vertebrates worldwide [1], require urgent research and protection efforts. Among them, the salamandrid genus *Pachytriton* Boulenger, 1878, endemic to eastern and southeastern China, has attracted significant taxonomic interest. To date, ten nominal species have been recorded for the genus *Pachytriton*, including: *P. brevipes* Sauvage, 1876, *P. granulosus* Chang, 1933, *P. archospotus* Shen, Shen and Mo, 2008, *P. inexpectatus* Nishikawa, Jiang, Matsui and Mo, 2011, *P. feii* Nishikawa, Jiang and Matsui, 2011, *P. moi* Nishikawa, Jiang and Matsui, 2011b, *P. changi* Nishikawa, Matsui and Jiang, 2012, *P. xanthospilos* Wu, Wang, and Hanken, 2012, *P. wuguanfui* Yuan, Zhang and Chen, 2016, and *P. airobranchiatus* Li, Yuan, Li and Wu, 2018. Notably, taxonomic consensus on the validity of *P. xanthospilos* and *P. changi* remains incomplete. Nishikawa et al. (2013) [2] synonymized *P. xanthospilos* Wu, Wang, and Hanken, 2012 with *P. changi*, treating the former as a junior synonym of the latter. However, the authoritative database Amphibians of the World (https://amphibiansoftheworld.amnh.org/Amphibia/Caudata/Salamandridae/Pleurodelinae/Pachytriton, accessed on 1 August 2025) still recognizes both *P. xanthospilos* and *P. changi* as valid species to date. To avoid potential ambiguity arising from analyzing a taxon with unresolved taxonomic status, only data for *P. changi* were incorporated into the morphological and phylogenetic analyses of this study, with no samples or data attributed to *P. xanthospilos* included.

Despite the ongoing global biodiversity crisis, amphibian diversity in China continues to be revealed at a remarkable pace. Notably, 44 new amphibian species (or new records) were described in 2022, accounting for 36.66% of newly added vertebrates that year [3], followed by 31 new additions in 2023, representing 33.33% of the annual vertebrate discoveries [4]. These findings highlight the necessity of comprehensive field surveys across under-explored regions, integrated with morphological and molecular analyses, to elucidate taxonomic boundaries and intra-specific variation within cryptic species complexes.

During herpetological surveys in the Qingliangfeng Nature Reserve (She County, Huangshan City, Anhui Province, China), we collected several specimens of *Pachytriton* from a remote montane habitat. Based on our preliminary literature survey, only two *Pachytriton* species, *P. granulosus* and *P. feii*, have been documented in Qingliangfeng National Nature Reserve and its surrounding areas [5,6,7]. Therefore, we simultaneously collected samples of *P. granulosus* and *P. feii* as reference taxa for comparative analyses. Superficially, these newly collected specimens resemble *P. granulosus* and *P. feii*. However, detailed morphological examinations and molecular phylogenetic analyses based on mitochondrial (*ND2*, *cytb*) and nuclear (*RAG1*, *POMC*) DNA sequences revealed that these individuals represent a distinct evolutionary lineage, inconsistent with all currently recognized congeners. Although the full distribution range of this taxon remains uncertain due to limited material, we herein describe it as a new species based on robust diagnostic characters and genetic divergence. The deposition of complete sequence data in GenBank will facilitate future identification and conservation efforts for this newly recognized lineage.

## 2. Materials and Methods

### 2.1. Sampling

**Figure 1:**
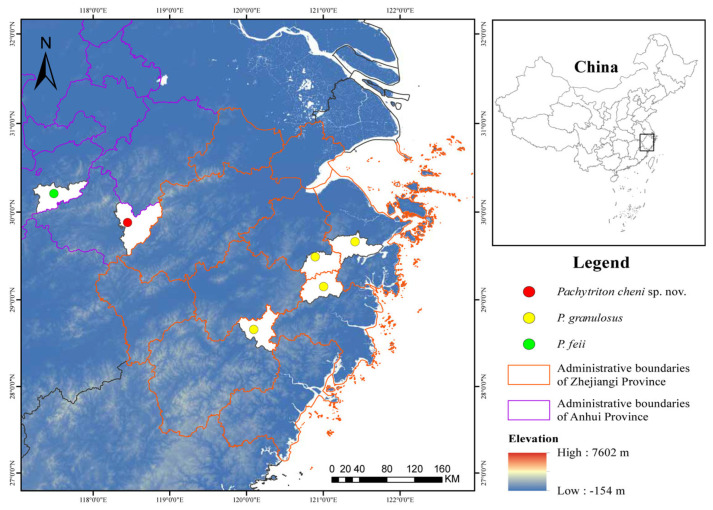
Map of sampling localities for *Pachytriton cheni* sp. nov., *P. granulosus*, and *P. feii*.

Field surveys targeting the genus *Pachytriton* were conducted in Anhui and Zhejiang Provinces, China, in August and September 2023 (Figure 1). A limited number of specimens were collected for each species due to their restricted distributions and preference for remote, challenging habitats, which complicates sampling efforts. In total, 14 *Pachytriton* specimens were obtained (Table 1). These included four individuals of an undescribed species from Qingliangfeng Nature Reserve, She County, Huangshan City, Anhui Province; five individuals of *P. feii* from Shitai County, Chizhou City, Anhui Province; and five individuals of *P. granulosus* from Shaoxing, Ningbo, Taizhou, and Lishui Cities in Zhejiang Province.

Sex was determined based on the presence of a significantly swollen and prominent cloaca. Specimens were humanely euthanized via lethal injection of a 0.7% tricaine methanesulfonate (MS-222, Changmao Biochemical Engineering Co., Ltd., Changzhou, China) solution. Fresh liver tissue was dissected and immediately preserved in 95% ethanol. Whole specimens were fixed in 10% formaldehyde for 24 h and subsequently transferred to 75% ethanol for permanent preservation. All voucher specimens were deposited in the Museum of Anhui Normal University (ANU). Sampling procedures involving live animals complied with China’s Wild Animals Protection Law and were approved by the Institutional Ethics Committee of Anhui Normal University (Protocol Code: AHNU-ET2023056).

### 2.2. Molecular Data and Phylogenetic Analysis

Genomic DNA was extracted from preserved liver tissues using the TIANamp Genomic DNA Kit (Tiangen Biotech Co., Ltd., Beijing, China). We performed phylogenetic analyses on 39 *Pachytriton* specimens using mitochondrial genes (*ND2* and *cytb*) and nuclear genes (*RAG1* and *POMC*), all of which are established markers for this genus [10]. The *ND2* gene was amplified with primers ND2-4F/ND2-4R [13] under the following PCR conditions: 95 °C for 4 min; 35 cycles of 95 °C for 40 s, 53 °C for 34 s, and 72 °C for 60 s; final extension at 72 °C for 10 min. For *cytb*, we used primers 14590F/15293R [9] with cycling parameters of 94 °C for 2 min; 35 cycles of 94 °C for 30 s, 52 °C for 45 s, and 72 °C for 90 s; and a final extension at 72 °C for 5 min. Nuclear *RAG1* was amplified using primers 1738F/3049R [9] under identical conditions to *cytb* except for an increased annealing temperature (56 °C) and extended final elongation (7 min). Similarly, *POMC* amplification employed degenerate primers *POMC*_DRV_F1/R1 [14] with the same protocol as *RAG1*. The PCR products were sequenced using an ABI 3730 XL Genetic Analyzer (Applied Biosystems, Foster City, CA, USA) at Sangon Biotech Co., Ltd. (Shanghai, China). The raw sequence data reported in this paper have been deposited in the Genome Se-quence Archive [15] in National Genomics Data Center [16], China National Center for Bioinformation / Beijing Institute of Genomics, Chinese Academy of Sciences (GSA: CRA031206) that are publicly accessible at https://ngdc.cncb.ac.cn/gsa, accessed on 10 October 2025.

This study integrates newly generated sequence data (accessible via the GSA ac-cession CRA031206) with published sequences sourced from the National Center for Biotechnology Information (NCBI) GenBank database. Phylogenetic analyses included all currently recognized species of the genus *Pachytriton*, with four sequences of *Paramesotriton* Chang, 1935 employed as the outgroup (Table 1). The *ND2* and *cytb* genes were concatenated into a single contiguous sequence. All sequences were aligned using the Clustal W algorithm with default parameters [17], followed by trimming via the partial gap deletion option in MEGA 12 [18]. Subsequently, the mean uncorrected interspecific and intraspecific *p*-distances were computed. The optimal nucleotide substitution model was determined using jModelTest 2.1.6 [19] based on the Akaike Information Criterion, and the GTR + G model was identified as the most suitable for the data in this study. Bayesian Inference (BI) analysis was carried out using MrBayes 3.2.7 [20], while Maximum Likelihood (ML) analysis was conducted in MEGA 12. For the BI analysis, two independent runs were configured, each involving 10,000,000 generations of Markov chains and sampling every 1000 generations. After assessing chain convergence in Tracer v1.6 [21], the first 25% of the trees were discarded as burn-in. For the ML analysis, a bootstrap consensus tree was constructed from 1000 replicates. For the nuclear genes *RAG1* and *POMC*, heterozygous sites were identified, and haplotypes were statistically inferred using DnaSP v5 [22]. Finally, median-joining haplotype networks were reconstructed in PopART [23] to visualize genetic relationships.

Following the methodological approach of Wu et al. (2015) [24], we integrated statistical species delimitation into the species discovery process. To rigorously test whether the Qingliangfeng population constitutes an independent evolutionary lineage, we employed Bayes factors to evaluate competing species delimitation hypotheses under the Multispecies Coalescent Model (MSC) [25,26]. The MSC provides a robust statistical framework for species delimitation by explicitly modeling the stochasticity of gene tree histories within a species tree, thereby accounting for incomplete lineage sorting (ILS), which is a major source of gene tree-species tree discordance [27]. This approach is particularly powerful for delimiting recently diverged species or taxa with complex evolutionary histories [28,29]. Within the MSC framework, we constructed two alternative models corresponding to the competing hypotheses: (1) the Qingliangfeng population is conspecific with *P. granulosus* (a one-species model), and (2) the Qingliangfeng population is a distinct species from *P. granulosus* (a two-species model). The Bayesian factor comparison was then used to assess which model was better supported by our multi-locus dataset (combining mitochondrial *ND2* and *cytb*, and nuclear *RAG1* and *POMC* sequences). This method of using Bayes factors to compare MSC-based models is an established strategy for determining taxonomic status [28]. Samples with missing data for more than two of the four genes were excluded from this analysis to ensure data quality. The analysis was implemented using the *BEAST module in BEAST v2.6.7 [25,30], which co-estimates species trees and species boundaries under the MSC. Marginal model likelihoods for each delimitation hypothesis were calculated using both path sampling (PS; [31]) and stepping-stone sampling (SS; [32]) methods. The population size model was set to piecewise linear with a constant root, and the population size parameter (θ) was assigned an inverse gamma prior (α = 4, β = 0.003; mean = 0.001). The Markov chains were run for 50 million generations, sampling every 5000 generations. Marginal likelihood estimation involved 100 path steps, each running for 1 million generations (totaling 100 million generations). Bayes factors (2lnBF) were calculated and interpreted following the criteria of Kass and Raftery (1995) [33]: 2lnBF > 2 indicates “positive support,” >6 denotes “strong support,” and >10 represents “decisive support” for the better model.

### 2.3. Morphological Comparison and Analyses

Morphological analyses followed the methodology outlined in Wu et al. (2012) [34]. Externally measured data of newly collected specimens were obtained using a digital caliper (Deli DL91200, Deli Group Co., Ltd., Ningbo, Zhejiang, China) with a precision of 0.1 mm. Measured morphological traits and their abbreviations are provided in Table 2. For symmetric cephalic characters, only the right side was measured; for asymmetric characters, measurements were taken from both sides, and the mean value was calculated. Morphological traits of the remaining *Pachytriton* species for comparative analyses were sourced from key references [11,34,35,36]. To account for the confounding effects of allometric growth, we followed the method of Thorpe (1975) [37] and size-corrected each morphometric trait by expressing it as a ratio relative to snout-vent length (SVL) (R, %).

Statistical analyses of morphometric data from the unnamed specimens, *Pachytriton granulosus*, and *Pachytriton feii* were conducted in R version 4.3.2, following the approach of Lyu et al. (2020) [38]. Given the obvious sexual size dimorphism [39], males and females were analyzed separately. All measurements were natural log-transformed to standardize data and reduce variance. One-way analysis of variance (ANOVA) was performed using the car package after verifying homoscedasticity (Levene’s test, *p* > 0.05). Principal Component Analysis (PCA) was implemented via the prcomp function and ggplot2 package to reduce the dimensionality of data variation and determine whether morphological variation constitutes a basis for distinguishable population structure.

## 3. Results

### 3.1. Molecular Phylogenetic and Species Delimitation Analyses

**Figure 2:**
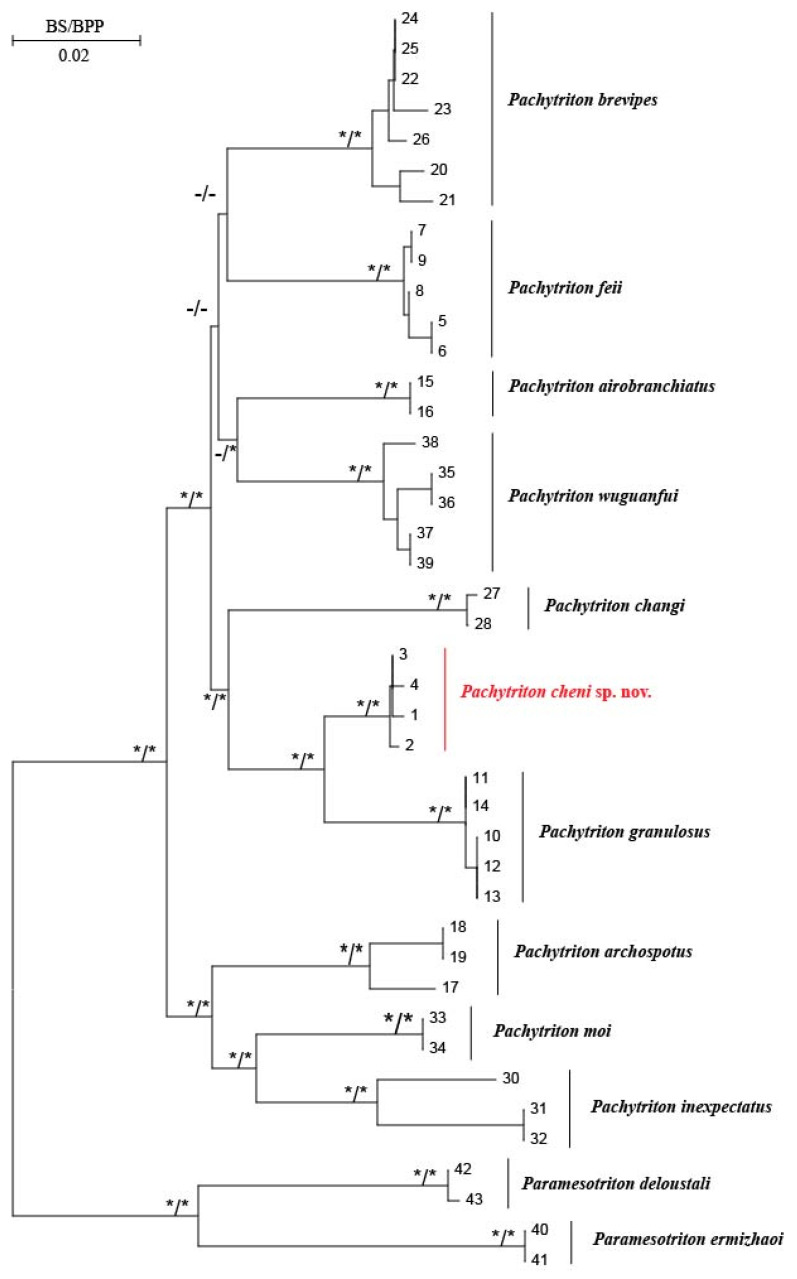
Bayesian tree and maximum likelihood tree constructed based on mitochondrial *ND2* gene and *cytb* gene, respectively. Asterisk denotes high support by Bayesian posterior probabilities (BPP > 95%) and bootstrap support values (BS > 70%). Long dash represents low support values. The new species, *Pachytriton cheni* sp. nov., is highlighted in red. ID number in Table 1 is displayed at the end of the lineages.

**Figure 3:**
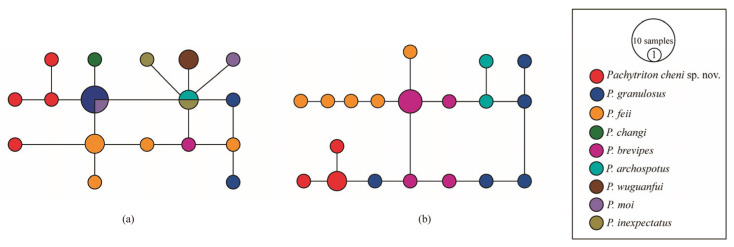
Haplotype networks of *Pachytriton cheni* sp. nov. and its related species constructed based on the nuclear gene sequences. (**a**) Haplotype network constructed from the nuclear gene POMC; (**b**) Haplotype network constructed from the nuclear gene RAG1. Different species of *Pachytriton* are shown in different colors.

Phylogenetic analyses of the mitochondrial *ND2* and *cytb* genes using Bayesian Inference (BI) and Maximum Likelihood (ML) produced highly congruent topologies, with strong support for all major nodes (Figure 2). These gene tree structures were consistent with those reported in previous studies [10,24,36]. Notably, in both BI and ML analyses, the four specimens collected from Qingliangfeng Nature Reserve (She County, Huangshan City, Anhui Province) formed a well-supported monophyletic clade, which was recovered as the sister group to *Pachytriton granulosus* with robust node support. Analysis of the concatenated mitochondrial dataset revealed that the mean uncorrected pairwise *p*-distances between this population and other *Pachytriton* species ranged from 4.39% to 10.22% (Table 3), a range that not only falls within the currently recognized interspecific distance scope but also exceeds the interspecific divergences observed among other *Pachytriton* species. Additionally, haplotype networks constructed based on *RAG1* and *POMC* sequences showed that the population from Qingliangfeng Nature Reserve possessed unique haplotypic characteristics, allowing clear separation from congeners in the haplotype networks (Figure 3).

Given the sister relationship revealed by phylogenetic analyses, we employed Bayes factors within the Multispecies Coalescent Model (MSC) framework to evaluate two competing species delimitation hypotheses between the Qingliangfeng population and its closest relative, *Pachytriton granulosus*: (1) the Qingliangfeng population represents a geographic variant of *P. granulosus* (a one-species model), and (2) it constitutes a distinct species (a two-species model). The Bayes factor comparison of these MSC-based models, implemented using the Path Sampling (PS) method, provided decisive support for the two-species model (2lnBF = 24.52, Table 4) [33], thus strongly rejecting the hypothesis of conspecificity. The Stepping Stone (SS) method produced similar results, but we report only the PS results as it is generally considered more efficient [32]. However, the available genetic data are insufficient to test for conspecificity between the Qingliangfeng population and either *P. airobranchiatus* or *P. changi*. Morphologically, the Qingliangfeng population in She County differs from both *P. airobranchiatus* and *P. changi* in whether the tips of the digits contact when the limbs are adpressed (see Comparisons below). Furthermore, based on mitochondrial divergence, it is unlikely that the Qingliangfeng population is conspecific with *P. airobranchiatus* or *P. changi*. Therefore, the population from the Qingliangfeng Nature Reserve in She County should be considered an independently evolving lineage within the genus *Pachytriton* and thus warrants description as a new species.

### 3.2. Morphological Comparisons

**Figure 4:**
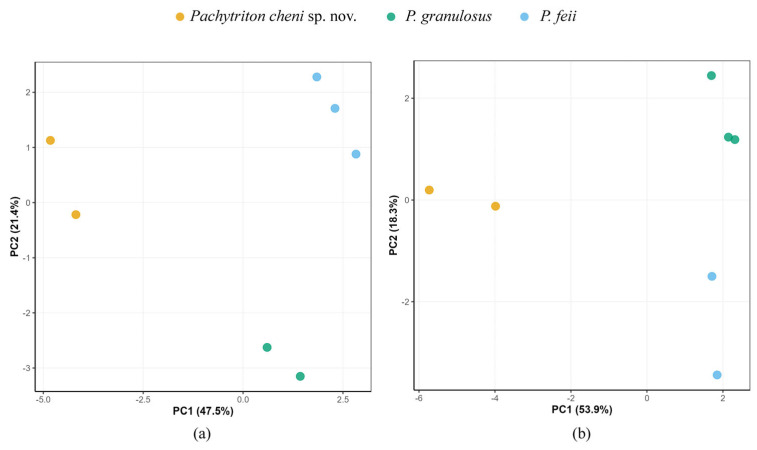
Scatter plot of PC1 and PC2 of Principal Component Analysis based on the morphometric measurements, distinguishing the samples of *Pachytriton cheni* sp. nov., *P. granulosus,* and *P. feii*. (**a**) Male; (**b**) Female.

Morphologically, the population from Qingliangfeng National Nature Reserve in She County can be clearly distinguished from all known congeners (see taxonomic account below). Statistical analyses of morphological measurements were conducted on populations of *Pachytriton* from Qingliangfeng National Nature Reserve, *P. feii* from Shitai County in Chizhou City, and *P. granulosus* from Zhejiang and Anhui (Table 5; Figure 4).

Compared with male *P. granulosus*, males of the She County population exhibit significantly smaller RUEW and RBTAW (*p*-values < 0.05), but significantly larger RVL (*p*-values < 0.05). Females of the She County population have significantly smaller RHW, RENL, RUEW, ROL, and RMXTAH (*p*-values < 0.05), while possessing significantly larger RHL, RIOD, and RVL (*p*-values < 0.05) compared with female *P. granulosus*. Relative to male *P. feii*, males of the She County population show significantly larger RIOD, RVL, and RMTAW (*p*-values < 0.05), but significantly smaller RIND, RUEW, RFLL, and RHLL (*p*-values < 0.05). Females of the She County population differ from female *P. feii* by having significantly smaller RHW and RENL (*p*-values < 0.05).

Principal component analysis (PCA) further supports the distinctiveness of the She County population. For males, the first two principal components (PCs) accounted for 68.9% of the total variance (PC1: 47.5%; PC2: 21.4%). PC1 was primarily loaded by traits such as ROL, RVL, RUEW, and RHW, while PC2 was mainly influenced by RIND and RBTAW. For females, the first two PCs explained 72.2% of the variance (PC1: 53.9%; PC2: 18.3%). PC1 was largely associated with RHW, RSL, ROL, and RUEW, whereas PC2 was primarily defined by SVL and RHLL. Scatter plots based on PC1 and PC2 (Figure 4) revealed clear separation of both male and female individuals of the She County population from *P. granulosus* and *P. feii*.

Combining the above morphological statistical analyses with the results of phylogenetic inference, we confirm that the *Pachytriton* population from She County, Huangshan City, represents a new species, which is described herein.

### 3.3. Taxonomic Accounts

*Pachytriton cheni* sp. nov.

https://zoobank.org/urn:lsid:zoobank.org:pub:156A8B05-91A9-4EFC-9283-C9419385D31B (accessed on 10 October 2025).

Holotype

**Figure 5:**
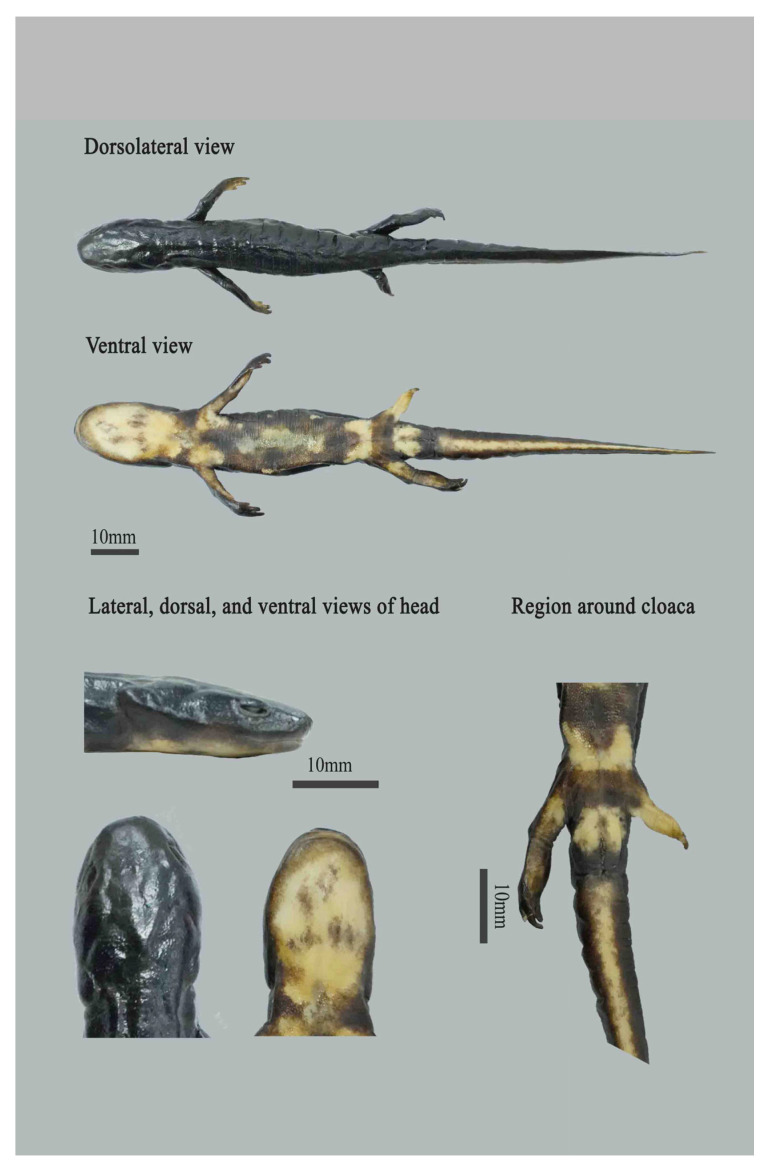
Holotype (ANU 20230001) of *Pachytriton cheni* sp. nov. in preservative. Credit: Siyu Wu.

ANU20230001 (Figure 5), an adult male, collected by Zhirong He and Siyu Wu on 24 August 2023 at a mountain stream at an altitude of 950 m in Qingliangfeng Nature Reserve (30.08445491° N, 118.86914777° E), She County, Huangshan City, Anhui province, China. When collecting, the depth of the mountain stream water flow is shallow, but the flow velocity is fast.

Paratype

A total of three specimens were collected by Zhirong He and Siyu Wu from Qingliangfeng Nature Reserve, She County, Huangshan City, Anhui Province, China. ANU20230002, an adult female, was collected under the same conditions as the holotype; ANU20230003, an adult male, was collected on 24 August 2023 from a mountain stream at an altitude of 1050 m; ANU20230004, a female individual, was collected on 27 September 2023 from a mountain stream at an altitude of 1300 m.

Etymology

The species is named in honor of the late Professor Bi-Hui Chen (1931–2022), a world-renowned herpetologist who dedicated his life to vertebrate education and research. Celebrated as the “father of the Chinese alligator”, Professor Chen decoded the survival strategies of the critically endangered *Alligator sinensis* through persistent field studies. His work was pivotal in developing effective conservation breeding programs that ultimately rescued the species from the brink of extinction. He also co-authored the seminal work “Anhui Amphibians and Reptiles Fauna” [40], which has profoundly contributed to herpetological studies in Anhui Province and across China. We name this new species *Pachytriton cheni* in his memory, proposing “Chen’s Stout Newt” as its English common name and “陈氏肥螈 (chén shì féi yuán)” as its Chinese common name.

Diagnosis

(1) A small-sized newt of the genus *Pachytriton*, males 57.1–69.1 mm and females 63.9–68.7 mm SVL; (2) head oval and narrow, head length greater than head width; (3) nearly black dorsal ground coloration lacking both bright orange spots and black speckling; (4) smooth skin texture; (5) the occipital region typically exhibits a distinct V-shaped ridge; (6) the abdomen is orange-red with several short brown streaks or vermiculate spots; (7) significant neck folds; (8) when adpressed, the digits of forelimbs and hindlimbs approach each other with minimal intervening space.

Description of the holotype

ANU20230001 (Figure 5), adult male with a slender and small-sized body (SVL 57.1 mm). Skin smooth; head oval in dorsal view and nearly flat in profile; snout truncate, protruding slightly beyond lower jaw; nostrils close to snout tip; parotoid region evident; cloacal opening oval, slightly protruding; four fingers and five toes, slender and elongated, lacking webbing; finger formula: 3 > 2 > 4 > 1; Toe style: 3 > 4 > 2 > 5 > 1; tail laterally compressed and the end is blunt and round.

Coloration

**Figure 6:**
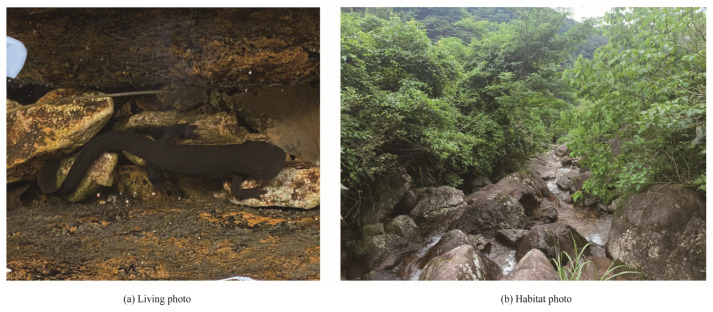
(**a**) *Pachytriton cheni* sp. nov. in life; (**b**) habitat at the *Pachytriton cheni* sp. nov. in Qingliangfeng Nature Reserve, Huangshan, Anhui, China. Credit: (**a**) Photo by Supen Wang; (**b**) Photo by Zhirong He.

In life (Figure 6), dorsum, flanks, limbs, and upper side of tail uniformly black; vertebral ridge uniformly black as dorsal surface; the abdomen is orange red in color, with a few brown short lines or worm-like spots; cloacal opening and underside of tail bright orange. In preservative (Figure 5), dorsum, flanks, and limbs uniformly dark. Ventral bright markings fading to cream.

Variations

Measurements of the type series are given in Table 5. Females (SVL 63.9–68.7 mm) distinctly larger than males (SVL 57.1–69.1 mm). The cloaca is wider and more swollen in males than in females; irregular bright orange patches on ventral surface vary among individuals. Compared with the holotype, all paratypes have fewer bright orange spots around the chin, abdomen, and cloaca; but in other forms, it is very similar to the holotype.

Distribution and habitat

From early August to late September, all individuals were gradually observed in a mountain stream on the mountaintop. *Pachytriton cheni* sp. nov. is currently known only from its type locality in Qingliangfeng Nature Reserve in southern Anhui (Figure 6). This new species inhabits small montane streams (1–2 m wide) in broadleaf forests near the top of the mountain at elevations ranging from 850 m to 1350 m. Large boulders are scattered throughout the stream. The flowing water is very clear, and some areas have formed small ponds after the rain. During the investigation, the water temperature was between 17.5 °C and 18.9 °C. The pH value of water was close to 7.6 according to pH meter. Stream substrates include gravels, scattered small rocks, leaves, and sands. The surrounding forests are mainly composed of *Emmenopterys henryi* Oliv., *Alnus rubra* Bong., *Pseudolarix amabilis* (J. Nelson) Rehder, *Quercus stewardii* Rehder, and *Phyllostachys edulis* (Carrière) J. Houz. Other amphibians and reptiles that co-inhabit the stream include *Amolops wuyiensis*, *Quasipaa exilispinosa*, *Trimeresurus stejnegeri,* and *Deinagkistrodon acutus*.

Comparisons

*Pachytriton cheni* sp. nov. is unambiguously assigned to the genus *Pachytriton* based on its morphological characters and phylogenetic position. The new species can be readily distinguished from all recognized congeners by a suite of unique and discrete morphological characteristics supported by statistical analyses.

**Figure 7:**
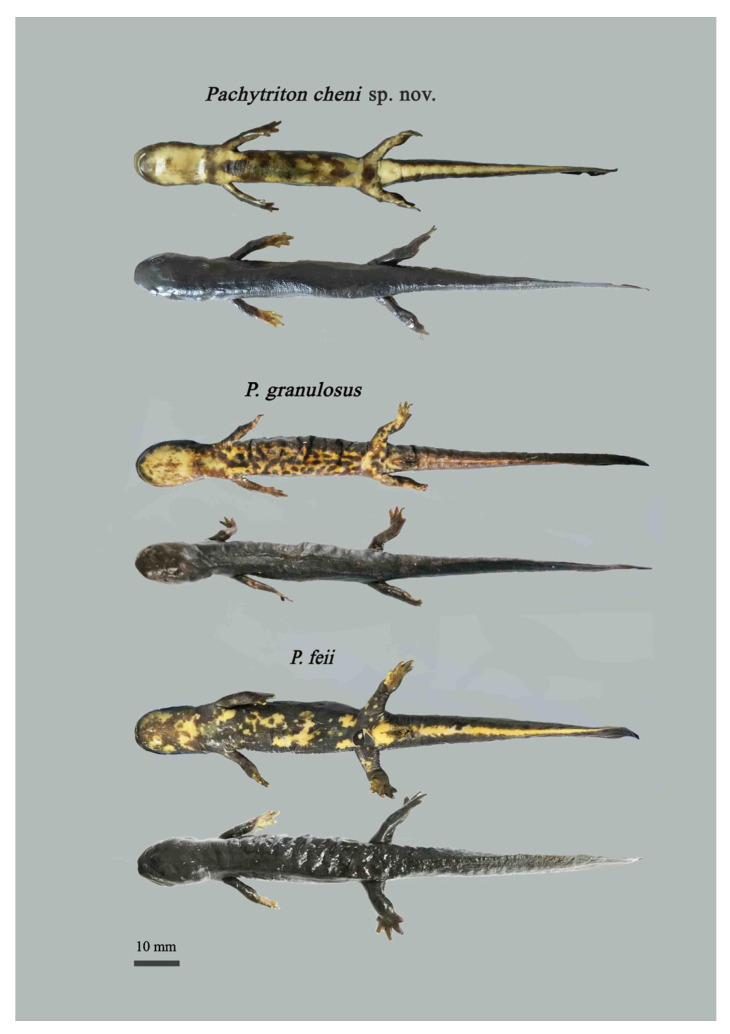
Comparison photos of the morphology of *Pachytriton cheni* sp. nov., *P. granulosus,* and *P. feii*. Credit: Siyu Wu.

Compared to its sister species *P. granulosus* (Figure 7), *Pachytriton cheni* sp. nov. is distinguishable by a suite of integrated morphological traits that reflect consistent differences in cranial, caudal, and trunk morphology. Cranially, the new species exhibits a more slender head (evidenced by 8.2–25.2% smaller relative head width [RHW] in both sexes) paired with reduced ocular structures—specifically, eyes that are 54.8–54.9% smaller in orbital length (ROL) and upper eyelids that are 61.8–61.9% narrower (RUEW) than those of *P. granulosus*. Caudally, it possesses a proportionally more elongated tail (9.2–12.2% longer relative tail length [RTAL] across sexes) with a distinctly slender base (26.0% narrower relative base tail width [RBTAW] in males), creating a more streamlined caudal profile compared to the thicker, shorter-tailed *P. granulosus*. Trunk differences include a 5.8–5.9% greater axilla–groin distance (RAGD) in males, contributing to a less compact body shape. Qualitatively, these structural differences are complemented by the new species’ distinct occipital V-shaped ridge (weak or absent in *P. granulosus*) and unspotted dorsum (vs. frequent orange-red dorsal spots in *P. granulosus*), as well as minimal spacing between adpressed limb digits (vs. a noticeable gap in *P. granulosus*).

In contrast to *P. feii* (Figure 7), *Pachytriton cheni* sp. nov. displays key morphological divergences in both structural proportions and color patterning. Most notably, the new species has a more delicate cranial and appendicular morphology: its head is 12.7–24.1% narrower (RHW) in both sexes, and males exhibit 15.2% shorter forelimbs (RFLL) and 15.7% shorter hindlimbs (RHLL) than male *P. feii*, reflecting a shift toward a less robust body plan. Caudally, it differs in tail architecture—possessing a 6.9–15.4% longer tail (RTAL) with a 45.1% wider mid-tail width (RMTAW) in males, resulting in a longer, more laterally expanded tail compared to the thinner, shorter tail of *P. feii*. Qualitative traits further distinguish the two species: the new species has a prominent occipital V-shaped ridge (less pronounced in *P. feii*) and minimal spacing between adpressed limb digits (vs. a 1–1.5 costal fold gap in *P. feii*). Ventrally, the new species’ orange-red abdomen with brown streaks/vermiculate spots contrasts sharply with *P. feii*’s pale ventral surface, which bears scattered, faint orange-red/orange-yellow patches (particularly reduced in adults).

When compared to *P. changi*, *P. wuguanfui*, and *P. inexpectatus*, the new species exhibits differences in pedal morphology. Specifically, it possesses a relatively shorter first finger and first toe, along with less developed interdigital webbing.

Unlike *P. airobranchiatus*, the new species lacks the prominent lateral head ridges characteristic of that species.

In comparison to both *P. brevipes* and *P. archospotus*, the new species completely lacks the extensively distributed black spots on the dorsum, while the two species have them.

As for *P. moi*, the new species exhibits a smaller body size (based on SVL), a narrower head, and a proportionally longer tail.

Conservation recommendation

Due to the Qingliangfeng Nature Reserve being located in the southern part of China, the *Pachytriton* genus is generally suitable for cold water [34], while the high annual temperature restricts the distribution of the new species to high elevations near the top of the mountain. Individuals of this newt sometimes gather together in deeper pools, but its population size seems much smaller compared to other more common species, such as *P. feii* and *P. granulosus*. Even though the type locality of *Pachytriton cheni* sp. nov. is well protected by the Qingliangfeng Nature Reserve and no major threat factors have been observed, the extent of occurrence of this species is estimated to be less than 100 km^2^, and the area of occupancy is estimated to be less than 10 km^2^. This new species belongs to *Pachytriton* in amphibians, which has been facing severe survival pressure recently. Luedtke et al. (2023) [1] integrated data from GAA1 and the Second Global Amphibian Assessment (GAA2) in 2022 and pointed out in the journal Nature that the pathogen of chytrid is gradually becoming a new threat to amphibians. The main chytrid that infect amphibians include *Batrachochytrium dendrobatidis* (Bd) [41] and *B. salamanderivorans* (Bsal) [42]. If this new species is infected with Bd and Bsal, it will pose a huge threat to its population size [43,44]. In this study, we also conducted screening for Bd and Bsal. All the new species samples we collected were not infected with Bd and Bsal.

## 4. Discussion

The discovery of *Pachytriton cheni* sp. nov. from the montane streams of Qingliangfeng Nature Reserve provides significant insights into the speciation processes and biodiversity patterns within the genus *Pachytriton* in southeastern China. This region, characterized by its complex topography and climatic history, has long been recognized as a center of amphibian endemism [10,45]. While two congeneric species, *P. feii* and *P. granulosus*, have been previously documented in Anhui Province [7,46], our findings reveal that the remote high-elevation ecosystems of the Qingliangfeng Mountains harbor additional evolutionary lineages that have remained undetected until now. The pristine mountain streams in this under-explored area present ideal conditions for cryptic speciation events, particularly among stream-adapted salamanders with limited dispersal capabilities [47].

Comprehensive molecular analyses provide compelling evidence for the recognition of the Qingliangfeng population as a distinct species. Phylogenetic reconstruction based on both mitochondrial and nuclear DNA sequences consistently recovered *Pachytriton cheni* sp. nov. as a well-supported monophyletic clade sister to *P. granulosus*. The significant genetic distances (4.39–10.22% for mitochondrial genes) between *Pachytriton cheni* sp. nov. and other described *Pachytriton* species exceed typical intraspecific variation observed within the genus [24,40]. Most notably, Bayesian species delimitation analyses decisively rejected the hypothesis that the Qingliangfeng population represents a geographical variant of *P. granulosus* (2lnBF > 10), providing robust statistical support for its status as an independent evolutionary lineage [33]. The distinct haplotypes identified in nuclear genes (*RAG1* and *POMC*) further provide strong evidence for reproductive isolation from its congeners.

The speciation pattern observed in *Pachytriton cheni* sp. nov. represents a classic case of allopatric divergence driven by altitudinal segregation and geographic isolation. The species exhibits a strikingly different elevational distribution compared to its sympatric congeners: while *P. granulosus* typically inhabits streams at 50–700 m and *P. feii* occurs at 400–930 m, *Pachytriton cheni* sp. nov. is restricted to high-elevation streams between 850 and 1350 m. This vertical stratification, reinforced by the topographic barriers of the Qingliangfeng Mountains, has effectively prevented gene flow and promoted independent evolutionary trajectories. Such altitudinal speciation patterns have been increasingly documented in montane amphibians across Southeast Asia [48], highlighting the importance of mountain systems as generators of biodiversity.

Morphological comparisons reveal consistent differences that align with the genetic evidence and likely reflect ecological adaptations to the unique high-elevation environment. The significantly narrower head width (RHW), smaller eyes (ROL, RENL), and narrower eyelids (RUEW) in *Pachytriton cheni* sp. nov. compared to both *P. granulosus* and *P. feii* may represent hydrodynamic adaptations for life in fast-flowing montane streams [49]. The longer tail (RVL) and greater axilla–groin distance provide enhanced propulsion and stability in turbulent waters [50], while the shorter limbs (RFLL, RHLL) potentially improve maneuverability among rocky substrates [51].

In accordance with the authoritative database Amphibians of the World (https://amphibiansoftheworld.amnh.org/Amphibia/Caudata/Salamandridae/Pleurodelinae/Pachytriton, accessed on 12 October 2024), which recognizes *Pachytriton xanthospilos* as a valid species, the genus *Pachytriton* now comprises 11 recognized species following the description of *Pachytriton cheni* sp. nov. This discovery underscores the ongoing nature of amphibian diversification in eastern China and highlights the need for continued surveys in remote montane areas. The Qingliangfeng Nature Reserve, with its ancient geological history and diverse microhabitats, represents a particularly important biodiversity refuge that has preserved unique evolutionary lineages [52]. The Huangshan region, identified as a Pleistocene refugium [19], continues to reveal unexpected biological diversity that contributes significantly to our understanding of regional biogeographic patterns.

From a conservation perspective, the restricted distribution of *Pachytriton cheni* sp. nov. and its specialization to a fragile high-elevation ecosystem warrant careful attention. Although the type locality is currently well-protected within the nature reserve, the species’ small population size and limited geographic range make it inherently vulnerable to environmental changes, including climate warming [53,54,55,56,57], habitat degradation [58,59], and emerging infectious diseases such as chytridiomycosis [1,60]. We recommend implementing long-term population monitoring programs and designating critical habitat zones specifically for this species within the reserve’s management plan.

## 5. Conclusions

This study describes *Pachytriton cheni* sp. nov., a new salamander species from the high-elevation streams of Qingliangfeng Nature Reserve in Anhui Province, China. Through integrative taxonomic analysis combining molecular phylogenetics and morphological comparisons, we demonstrate that this population represents a distinct evolutionary lineage that diverged from its closest relatives through allopatric speciation processes driven by altitudinal segregation and geographic isolation. The discovery of this species highlights the exceptional biodiversity value of southeastern China’s montane ecosystems and underscores the importance of continued field exploration in remote areas. It also emphasizes the utility of combining multiple lines of evidence in recognizing and validating cryptic species complexes among amphibians. As human impacts on natural ecosystems continue to intensify, documenting such evolutionary diversity becomes increasingly urgent for informing effective conservation strategies and understanding the historical processes that have shaped current biodiversity patterns in eastern Asia.

## Figures and Tables

**Table 1:** Voucher, localities, GenBank accession numbers, and source for all samples used in this study.

ID No.	Species Name	Voucher	Localities	GenBank No.				Source
				*ND2*	*cytb*	*RAG1*	*POMC*	
1	*Pachytriton cheni* sp. nov.	ANU20030001	China: Anhui: Huangshan: She	N/A	N/A	N/A	N/A	This study
2	*Pachytriton cheni* sp. nov.	ANU20030002	China: Anhui: Huangshan: She	N/A	N/A	N/A	N/A	This study
3	*Pachytriton cheni* sp. nov.	ANU20030007	China: Anhui: Huangshan: She	N/A	N/A	N/A	N/A	This study
4	*Pachytriton cheni* sp. nov.	ANU20030009	China: Anhui: Huangshan: She	N/A	N/A	N/A	N/A	This study
5	*Pachytriton feii*	ANU20030015	China: Anhui: Chizhou: Shitai	N/A	N/A	N/A	N/A	This study
6	*Pachytriton feii*	ANU20030016	China: Anhui: Chizhou: Shitai	N/A	N/A	N/A	N/A	This study
7	*Pachytriton feii*	ANU20030017	China: Anhui: Chizhou: Shitai	N/A	N/A	N/A	N/A	This study
8	*Pachytriton feii*	ANU20030018	China: Anhui: Chizhou: Shitai	N/A	N/A	N/A	N/A	This study
9	*Pachytriton feii*	ANU20030019	China: Anhui: Chizhou: Shitai	N/A	N/A	N/A	N/A	This study
10	*Pachytriton granulosus*	ANU20030022	China: Zhejiang: Shaoxing: Xingchang	N/A	N/A	N/A	N/A	This study
11	*Pachytriton granulosus*	ANU20030023	China: Zhejiang: Ningbo: Fenghua	N/A	N/A	N/A	N/A	This study
12	*Pachytriton granulosus*	ANU20030024	China: Zhejiang: Taizhou: Tiantai	N/A	N/A	N/A	N/A	This study
13	*Pachytriton granulosus*	ANU20030025	China: Zhejiang: Lishui: Jinyun	N/A	N/A	N/A	N/A	This study
14	*Pachytriton granulosus*	ANU20030026	China: Zhejiang: Lishui: Jinyun	N/A	N/A	N/A	N/A	This study
15	*Pachytriton airobranchiatus*	SWUFYZY0301	China: Guangdong: Huidong: Mt. Lianhua	MG732934	MG732932			[8]
16	*Pachytriton airobranchiatus*	SWUFYZY0213	China: Guangdong: Huidong: Mt. Lianhua	MG732933	MG732931			[8]
17	*Pachytriton archospotus*	CIB95953	China: Hunan: Guidong: Mt. Qiyun	GQ303628	GQ303665	GQ303706		[9]
18	*Pachytriton archospotus*	KIZ04564	China: Hunan: Guidong	KU375008	KU374979		KU375036	[9]
19	*Pachytriton archospotus*	CIB95949	China: Hunan: Guidong: Mt. Qiyun	GQ303630	GQ303667	GQ303708		[9]
20	*Pachytriton brevipes*	CIB95926	China: Jiangxi: Nanfeng: Mt. Junfeng	GQ303626	GQ303663	GQ303704		[9]
21	*Pachytriton brevipes*	CIB95930	China: Jiangxi: Nanfeng: Mt. Junfeng	GQ303627	GQ303664	GQ303705		[9]
22	*Pachytriton brevipes*	CIB88221	China: Fujian: Wuyi Shan: Mt. Wuyi	GQ303615	GQ303652	GQ303693		[9]
23	*Pachytriton brevipes*	CIB88194	China: Fujian: Wuyi Shan: Mt. Wuyi	GQ303616	GQ303653	GQ303694		[9]
24	*Pachytriton brevipes*	CIB88188	China: Fujian: Wuyi Shan: Mt. Wuyi	GQ303617	GQ303654	GQ303695		[9]
25	*Pachytriton brevipes*	CIB88197	China: Fujian: Wuyi Shan: Mt. Wuyi	GQ303618	GQ303655	GQ303696		[9]
26	*Pachytriton brevipes*	KIZ08928	China: Fujian: Wuyi Shan: Mt. Wuyi	KU375010	KU374981		KU375037	[10]
27	*Pachytriton changi*	KUHE 39832	Unknown locality		AB638711			[8]
28	*Pachytriton changi*	KUHE 39763	Unknown locality		AB638709			[11]
29	*Pachytriton changi*	KUHE:44985	China: Hunan: Nanling				LC746909	[12]
30	*Pachytriton inexpectatus*	KIZ08711	China: Hunan: Jiangyong	KU375031	KU375002		KU375044	[8]
31	*Pachytriton inexpectatus*	KIZ05203	China: Guangxi: Dayaoshan	KU375029	KU375000		KU375045	[8]
32	*Pachytriton inexpectatus*	KIZ05204	China: Guangxi: Dayaoshan	KU375030	KU375001			[8]
33	*Pachytriton moi*	KIZ07767	China: Guangxi: Maoershan	KU375032	KU375003			[8]
34	*Pachytriton moi*	KIZ07768	China: Guangxi: Maoershan	KU375033	KU375004		KU375046	[8]
35	*Pachytriton wuguanfui*	KIZ08756	China: Guangxi: Guposhan	KU375012	KU374983			[10]
36	*Pachytriton wuguanfui*	KIZ08761	China: Guangxi: Guposhan	KU375013	KU374984		KU375040	[10]
37	*Pachytriton wuguanfui*	KIZ021705	China: Hunan: Jiuweishan	KU375014	KU374985			[10]
38	*Pachytriton wuguanfui*	KIZ021706	China: Hunan: Jiuweishan	KU375015	KU374986		KU375041	[10]
39	*Pachytriton wuguanfui*	KIZ021707	China: Hunan: Jiuweishan	KU375016	KU374987			[10]
40	*Paramesotriton ermizhaoi*	CIB88141	China: Guangxi: Jin Xiu: Mt. Dayao	FJ744601	GQ303670			[9]
41	*Paramesotriton ermizhaoi*	CIB88140	China: Guangxi: Jin Xiu: Mt. Dayao	FJ744602	GQ303671			[9]
42	*Paramesotriton deloustali*	MVZ223628	Vietnam: Vinh Phu Province: Tam Dao: Vinh Yen District	FJ744599	GQ303668			[9]
43	*Paramesotriton deloustali*	MVZ223629	Vietnam: Vinh Phu Province: Tam Dao: Vinh Yen District	FJ744600	GQ303669			[9]

This study (samples 1–14): The complete dataset of raw sequences is available under GSA accession CRA031206. Individual gene accessions for comparative samples (15–43) are from NCBI GenBank. N/A, not applicable.

**Table 2:** Abbreviations and descriptions of morphological characteristics of adult individuals.

No.	Morphological Character	Abbreviation	Description
1	Snout-vent length	SVL	From tip of snout to anterior tip of vent
2	Head length	HL	From tip of snout to wrinkle of throat
3	Head width	HW	Measured at angle anterior to parotid grand
4	Maximum head width	MXHW	Measured at widest point
5	Snout length	SL	From tip of snout to anterior tip of upper eyelid
6	Eyelid-nostril length	ENL	Minimum distance between eyelid and nostril
7	Internarial distance	IND	Minimum distance between the external nares
8	Interorbital distance	IOD	Minimum distance between upper eyelids
9	Upper eyelid width	UEW	Greatest width of upper eyelid
10	Upper eyelid length	UEL	Greatest length of upper eyelid
11	Orbit length	OL	Maximum length of orbit
12	Axilla-groin distance	AGD	Minimum distance between axilla and groin
13	Trunk length	TRL	From wrinkle of throat to anterior tip of vent
14	Tail length	TAL	From anterior tip of vent to tail tip
15	Vent length	VL	From anterior to posterior tip of vent
16	Basal tail width	BTAW	Tail width measured at root of tail
17	Medial tail width	MTAW	Tail width measured at middle
18	Basal tail height	BTAH	Tail height measured at base of tail
19	Maximum tail height	MXTAH	Tail height measured at highest point
20	Forelimb length	FLL	Distance from axilla to tip of longest finger
21	Hindlimb length	HLL	Distance from groin to tip of longest toe

**Table 3:** Net mean uncorrected *p*-distances (%) for *Pachytriton* populations from Qingliangfeng National Nature Reserve and within the new species vs. other congeneric species, based on concatenated mitochondrial datasets (*ND2* and *cytb*). Bold values highlight genetic distances for *Pachytriton cheni* sp. nov.

ID	Species	1	2	3	4	5	6	7	8	9	10
1	*Pachytriton cheni* sp. nov.	0.39									
2	*Pachytriton granulosus*	4.39	0.12								
3	*Pachytriton feii*	6.48	7.55	0.35							
4	*Pachytriton archospotus*	8.11	9.93	7.25	1.50						
5	*Pachytriton brevipes*	6.54	7.52	5.30	8.41	0.76					
6	*Pachytriton wuguanfui*	6.31	8.36	7.14	7.84	6.59	0.51				
7	*Pachytriton airobranchiatus*	6.63	8.17	5.98	8.37	6.27	6.32	-			
8	*Pachytriton changi*	6.77	7.77	7.85	9.46	7.22	7.08	7.48	0.18		
9	*Pachytriton inexpectatus*	10.22	11.70	10.34	10.75	10.87	11.45	10.27	9.76	3.07	
10	*Pachytriton moi*	7.56	8.85	8.10	7.92	8.26	9.30	8.54	9.64	8.64	-

Diagonal values represent intragroup *p*-distances. “-” indicates genetic distances < 0.1%.

**Table 4:** Marginal likelihood estimates and Bayes factor species delimitation results for the Qingliangfeng populations of *Pachytriton*.

Competing Delimitations	Path Sampling
*P.* sp. (Qingliangfeng) conspecific to *P. granulosus*	−7335.6368
*P.* sp. (Qingliangfeng) independent from *P. granulosus*	−7323.3744
2lnBf	24.52

According to Kass and Raftery (1995) [33], under the multispecies coalescent (MSC) model, a Bayes factor value of 2lnBF > 10 indicates “decisive” support for the more optimal model—herein referring to the “independent species model” (i.e., the Qingliangfeng population represents a distinct species from *P. granulosus*).

**Table 5:** Measurements of types and other specimens of *Pachytriton* examined (means ± SD of SVL <in mm> and medians of ratios of characters <R: %SVL>, with ranges in parentheses). For character abbreviations, refer to Table 2. Only display the *p*-values of *Pachypriton cheni* sp. nov. and *P. granulatus*, as well as the *p*-values of *Pachypriton cheni* sp. nov. and *P. feii*. * *p*-values < 0.05, ** *p*-values < 0.01.

Species	*Pachytriton cheni* sp. nov.		*P. granulosus*		*P. feii*		*P. changi*	*P. wuguanfui*	*P. airobranchiatus*		*P. brevipes*	*P. archospotus*	*P. moi*	*P. inexpectatus*
Gender	Male (*n* = 2)	Female (*n* = 2)	Male (*n* = 2)	Female (*n* = 3)	Male (*n* = 3)	Female (*n* = 2)	Male (*n* = 2)	Female (*n* = 1)	Male (*n* = 4)	Female (*n* = 2)	Male (*n* = 2)	Male (*n* = 3)	Male (*n* = 1)	Male (*n* = 14)
SVL	63.1 ± 8.4 (57.1–69.1)	66.3 ± 3.4 (63.9–68.7)	74.0 ± 1.6 (72.9–75.2)	60.6 ± 4.8 (55.1–63.6)	84.0 ± 4.6 (78.9–87.7)	85.8 ± 9.0 (79.4–92.1)	83.0 ± 2.9 (81.8–84.2)	83.5	73.1 ± 30.8 (63.3–76.8)	66.1 ± 5.4 (64.4–67.7)	80.4 ± 105.1 (73.1–87.6)	86.8 ± 5.9 (81.5–93.1)	100.2	87.8 ± 10.1 (68.6–102.1)
RHL	30.0 ± 5.7 (26.0–34.0)	29.6 ± 0.8 (29.1–30.1)	26.8 ± 0.8 (26.2–27.4)	26.5 ± 1.1 (25.2–27.4) *	28.1 ± 2.5 (26.2–30.9)	25.1 ± 0.2 (25.0–25.2)	25.3 ± 0.02 (25.2–25.4)	24.2	23.0 ± 1.0 (21.3–23.9)	22.2 ± 0.7 (21.6–22.7)	24.9 ± 1.1 (24.1–25.6)	25.5 (23.5–29.4)	35.9	28.7 (24.6–31.6)
RHW	17.2 ± 0.6 (16.8–17.7)	14.2 ± 0.7 (13.7–14.7)	18.4 ± 0.2 (18.2–18.6)	19.0 ± 0.9 (18.0–19.7) *	19.7 ± 0.2 (19.5–19.8)	18.7 ± 1.0 (18.0–19.4) *	18.5 ± 0.2 (18.2–18.8)	19.5	19.7 ± 0.1 (19.4–20.3)	19.3 ± 0.1 (19.1–19.6)	19.3 ± 0.2 (19.0–19.6)	21.7 (21.7–22.2)	23.2	19.8 (18.5–21.7)
RMXHW	19.8 ± 1.4 (18.8–20.8)	17.9 ± 0.9 (17.3–18.5)	19.6 ± 0.8 (19.0–20.1)	19.5 ± 1.0 (18.4–20.1)	20.7 ± 1.3 (19.9–22.2)	19.4 ± 0.2 (19.3–19.5)	19.9 ± 0.7 (19.3–20.5)	-	-	-	21.2 ± 1.1 (20.4–21.9)	24.3 (23.8–24.6)	25.3	22.3 (20.7–25.2)
RSL	7.3 ± 0.3 (7.1–7.5)	6.1 ± 0.7 (5.6–6.6)	8.6 ± 0.7 (8.1–9.1)	8.4 ± 0.3 (8.2–8.8)	8.7 ± 0.2 (8.5–8.8)	8.4 ± 0.7 (7.9–8.9)	10.0 ± 0.0 (9.8–10.1)	9.2	10.2 ± 0.5 (9.5–11.1)	9.6 ± 0.0 (9.5–9.7)	8.7 ± 0.1 (8.4–8.9)	8.0 (7.8–8.3)	11.3	9.5 (8.6–10.6)
RENL	6.7 ± 0.1 (6.6–6.8)	5.6 ± 0.1 (5.5–5.7)	6.8 ± 0.0 (6.8–6.8)	6.9 ± 0.4 (6.5–7.3) *	6.7 ± 0.1 (6.6–6.8)	6.8 ± 0.0 (6.7–6.8) *	7.3 ± 0 (7.2–7.3)	-	-	-	6.6 ± 0.0 (6.6–6.6)	5.7 (5.4–5.9)	8.6	7.0 (6.4–7.9)
RIND	6.3 ± 0.0 (6.3–6.3)	7.0 ± 0.0 (7.0–7.0)	6.0 ± 0.1 (6.0–6.0)	5.8 ± 0.9 (4.8–6.3)	6.5 ± 0.1 (6.4–6.6) *	6.1 ± 0.2 (6.0–6.3)	7.0 ± 0.04 (6.8–7.1)	4.6	9.1 ± 4.3 (5.6–10.7)	9.5 ± 0.9 (8.9–10.2)	6.0 ± 1.0 (5.3–6.7)	5.0 (4.9–5.8)	7.5	6.5 (5.9–7.4)
RIOD	11.8 ± 0.6 (11.4–12.1)	11.2 ± 0.0 (11.2–11.3)	6.9 ± 0.9 (6.3–7.6)	7.1 ± 0.6 (6.5–7.5) **	8.6 ± 0.3 (8.4–8.9) *	7.1 ± 0.9 (6.5–7.7)	7.4 ± 0.3 (7.0–7.7)	9.2	10.7 ± 0.2 (10.2–11.4)	10.4 ± 0.1 (10.1–10.6)	7.2 ± 4.2 (5.7–8.6)	9.3 (8.8–9.6)	8.0	6.9 (6.4–7.9)
RUEW	0.9 ± 0.1 (0.8–1.0)	1.3 ± 0.0 (1.3–1.3)	3.4 ± 0.4 (3.1–3.6) *	3.4 ± 0.4 (3.0–3.8) **	2.8 ± 0.2 (2.6–3.0) *	2.6 ± 0.3 (2.4–2.9)	2.7 ± 0.1 (2.4–2.9)	-	-	-	2.7 ± 0.1 (2.4–2.9)	1.5 (1.2–1.6)	2.7	2.5 (2.0–3.2)
RUEL	4.8 ± 1.1 (4.1–5.6)	4.5 ± 1.0 (3.8–5.3)	5.9 ± 0.5 (5.5–6.3)	6.5 ± 0.3 (6.2–6.7)	5.2 ± 0.4 (4.7–5.5)	5.0 ± 0.1 (4.9–5.1)	4.7 ± 0.1 (4.5–4.9)	-	3.3 ± 0.7 (2.3–4.3)	3.9 ± 0.2 (3.5–4.2)	4.8 ± 0.0 (4.7–4.8)	3.8 (3.7–4.2)	4.7	4.9 (3.9–6.1)
ROL	1.4 ± 0.3 (1.2–1.6)	1.5 ± 0.1 (1.5–1.6)	3.1 ± 0.4 (2.9–3.4)	3.1 ± 0.4 (2.7–3.4) **	3.8 ± 0.2 (3.5–4.0)	3.1 ± 0.6 (2.6–3.5)	3.4 ± 0.3 (3.0–3.7)	-	-	-	3.4 ± 0.0 (3.3–3.5)	2.6 (2.1–2.8)	3.7	2.9 (2.4–3.5)
RAGD	54.6 ± 3.5 (52.2–57.1)	51.5 ± 6.0 (47.2–55.7)	51.6 ± 0.6 (51.2–52.0)	52.8 ± 0.9 (51.9–53.7)	48.4 ± 1.8 (46.6–50.2)	49.9 ± 0.0 (49.9–50.0)	50.1 ± 5.1 (48.5–51.7)	-	-	-	51.9 ± 0.1 (51.6–52.1)	50.1 (49.2–50.9)	52.1	49.5 (43.7–53.7)
RTRL	71.9 ± 3.1 (69.7–74.1)	72.1 ± 0.5 (71.7–72.5)	73.7 ± 1.5 (72.6–74.7)	73.4 ± 1.0 (72.3–74.2)	73.4 ± 1.1 (72.2–74.2)	74.7 ± 2.1 (73.3–76.2)	74.7 ± 0.0 (74.5–74.8)	-	-	-	75.2 ± 1.1 (74.4–75.9)	74.5 (70.6–76.5)	64.1	71.3 (68.4–75.4)
RTAL	110.1 ± 2.3 (108.5–111.7)	113.5 ± 5.9 (109.4–117.7)	101.7 ± 1.5 (100.6–102.7)	101.2 ± 2.2 (99.8–103.8)	102.9 ± 2.9 (99.8–105.6)	98.3 ± 3.7 (95.7–100.9)	103.1 ± 8.0 (101.1–105.1)	86.5	92.2 ± 44.3 (84.2–98.8)	93.4 ± 16.7 (90.5–96.3)	96.2 ± 49.0 (91.2–101.1)	95.7 (93.5–95.8)	90.5	90.6 (85.4–98.7)
RVL	11.8 ± 0.7 (11.3–12.2)	11.2 ± 0.3 (11.0–11.4)	7.9 ± 0.6 (7.5–8.3) *	6.7 ± 0.9 (5.7–7.4) *	7.3 ± 0.6 (6.7–7.8) **	4.3 ± 1.1 (3.6–5.1)	5.9 ± 0.1 (5.7–6.1)	-	-	-	6.2 ± 2.0 (5.2–7.2)	5.0 (4.8–5.0)	5.2	5.6 (4.6–7.5)
RBTAW	10.9 ± 0.3 (10.7–11.1)	10.8 ± 1.2 (9.9–11.6)	14.7 ± 0.2 (14.6–14.8) *	14.8 ± 0.6 (14.1–15.3)	12.3 ± 0.9 (11.4–13.2)	11.9 ± 1.4 (10.9–12.9)	12.1 ± 0.0 (12.0–12.2)	12.1	11.2 ± 0.7 (10.1–12.2)	12.2 ± 2.9 (11–13.4)	14.7 ± 0.3 (14.3–15.0)	15.8 (15.7–17.2)	13.2	14.7 (12.3–16.5)
RMTAW	13.2 ± 0.1 (13.2–13.3)	12.4 ± 0.8 (11.8–12.9)	11.6 ± 1.1 (10.8–12.3)	12.0 ± 1.0 (11.0–13.0)	9.1 ± 0.6 (8.4–9.6) *	8.7 ± 1.3 (7.8–9.6)	7.9 ± 0.3 (7.5–8.3)	-	-	-	10.9 ± 1.6 (10.0–11.8)	9.8 (8.6–12.5)	7.8	11.2 (8.9–13.5)
RBTAH	12.2 ± 1.4 (11.2–13.2)	12.2 ± 0.6 (11.7–12.6)	12.4 ± 1.1 (11.6–13.1)	12.6 ± 0.8 (11.7–13.3)	11.6 ± 0.6 (11.0–12.2)	11.4 ± 2.3 (9.8–13.1)	10.4 ± 0.3 (10.0–10.8)	8.4	10.4 ± 0.2 (10.2–11.1)	9.0 ± 1.4 (8.1–9.8)	13.2 ± 0.2 (12.9–13.5)	15.9 (14.0–16.0)	12.8	12.2 (10.3–14.2)
RMXTAH	14.1 ± 0.2 (13.9–14.3)	11.6 ± 0.1 (11.6–11.7)	16.2 ± 2.2 (14.7–17.8)	16.1 ± 1.2 (14.7–17.1) *	15.0 ± 0.5 (14.5–15.4)	14.9 ± 1.9 (13.6–16.3)	15.2 ± 0.1 (15.0–15.4)	-	-	-	20.3 ± 20.5 (17.1–23.5)	17.5 (16.1–18.0)	14.4	14.9 (12.4–15.7)
RFLL	22.3 ± 0.1 (22.3–22.4)	25.3 ± 0.6 (24.8–25.7)	22.5 ± 1.1 (21.8–23.2)	22.9 ± 0.4 (22.5–23.3)	26.3 ± 1.5 (24.6–27.4) *	24.3 ± 3.8 (21.6–27.0)	23.9 ± 0.7 (23.3–24.5)	17.6	15.3 ± 2.3 (13.8–17.8)	14.9 ± 9.0 (12.7–17)	20.7 ± 3.7 (19.3–22.0)	23.3 (21.7–24.3)	22.7	20.3 (18.5–23.6)
RHLL	24.6 ± 0.8 (24.0–25.1)	27.4 ± 0.6 (26.9–27.8)	24.9 ± 1.8 (23.6–26.1)	25.7 ± 1.4 (24.1–26.8)	29.2 ± 1.4 (27.7–30.4) *	29.4 ± 3.5 (26.9–31.9)	27.2 ± 0.9 (26.5–27.8)	20.7	17.1 ± 3.3 (14.7–19.6)	18.3 ± 0.2 (18–18.6)	25.4 ± 0.5 (24.9–25.9)	29.4 (25.9–29.6)	24.4	24.5 (21.8–35.3)

## Data Availability

The data that support the findings of this study have been deposited in the Genome Sequence Archive (GSA) at the National Genomics Data Center (NGDC), China National Center for Bioin-formation/Beijing Institute of Genomics, Chinese Academy of Sciences, under accession number CRA031206. These data are publicly accessible at https://ngdc.cncb.ac.cn/gsa, accessed on 10 October 2025. The published sequences from NCBI GenBank used in this study are available under the accession numbers provided in Table 1. Morphological specimens were deposited at Anhui Normal University (ANU), Wuhu, Anhui.
